# Influence of Menstrual Cycle or Hormonal Contraceptive Phase on Energy Intake and Metabolic Hormones—A Pilot Study

**DOI:** 10.3390/endocrines2020008

**Published:** 2021-04-16

**Authors:** Johanna K. Ihalainen, Ida Löfberg, Anna Kotkajuuri, Heikki Kyröläinen, Anthony C. Hackney, Ritva S. Taipale-Mikkonen

**Affiliations:** 1 Faculty of Sport and Health Sciences, University of Jyväskylä, 40014 Jyväskylä, Finland; 2 Department of Exercise & Sport Science-Department of Nutrition, University of North Carolina at Chapel Hill, Chapel Hill, NC 27599, USA; 3 Sports Technology Unit, Faculty of Sport and Health Sciences, University of Jyväskylä, 88610 Vuokatti, Finland

**Keywords:** sex hormones, estradiol, progesterone, energy availability, leptin, ghrelin

## Abstract

Sex hormones are suggested to influence energy intake (EI) and metabolic hormones. This study investigated the influence of menstrual cycle (MC) and hormonal contraceptive (HC) cycle phases on EI, energy availability (EA), and metabolic hormones in recreational athletes (eumenorrheic, NHC = 15 and monophasic HC-users, CHC = 9). In addition, 72-h dietary and training logs were collected in addition to blood samples, which were analyzed for 17β-estradiol (E2), progesterone (P4), leptin, total ghrelin, insulin, and tri-iodothyronine (T3). Measurements were completed at four time-points (phases): Bleeding, mid-follicular (FP)/active 1, ovulation (OVU)/active 2, mid-luteal (LP)/inactive in NHC/CHC, respectively. As expected, E2 and P4 fluctuated significantly in NHC (*p* < 0.05) and remained stable in CHC. In NHC, leptin increased significantly between bleeding and ovulation (*p* = 0.030) as well as between FP and OVU (*p* = 0.022). No group differences in other measured hormones were observed across the MC and HC cycle. The mean EI and EA were similar between phases, with no significant differences observed in macronutrient intake over either the MC or HC. While the MC phase might have a small, but statistically significant effect on leptin, the findings of the present study suggest that the MC or HC phase does not significantly alter ad libitum EI or EA in recreational athletes.

## 1. Introduction

Women of reproductive age are exposed to hormonal fluctuations during their menstrual cycle (MC) [1]. Sex hormones, such as 17β-estradiol (E2) and progesterone (P4), have broad effects on several body systems and functions that have physiological and behavioral consequences, including those that influence nutritional habits [2]. The mean energy intake (EI) is reported to be lowest before ovulation, when E2 is high, whereas the highest levels of EI have been observed during the luteal phase when P4 is increased [2]. These observations suggest that P4 may have appetite-enhancing effects that would lead to higher EI, whereas E2 may potentially inhibit appetite and, thus, EI [2]. The hormonal contraceptive (HC) use suppresses the fluctuation of endogenous sex hormones via a negative feedback on gonadotrophic hormones, resulting in relatively stable and low E2 and P4 concentrations [3]. Some earlier studies have reported that women using HCs consumed slightly more calories compared to non-users [4,5], while others have not observed differences in ad libitum EI [6]. To our knowledge, however, there are no studies on the effects of hormonal contraceptive cycle (HC), i.e., active hormonal versus inactive phase, on EI in recreational athletes. As HC use continues its upward trend among female athletes [7], it is important to consider the effects of varying concentrations of exogenous sex hormones on EI.

The dietary intake is modulated by the complex interplay of neurochemical, hormonal, physiological, and psychological factors. In this network, metabolic hormones, leptin, ghrelin, and insulin, play significant roles in appetite-regulation via specific neurons located in the hypothalamus [2]. Indeed, leptin, derived from adipose tissue, and insulin, from the pancreas, act as appetite-inhibiting signals that play an important role in long-term energy homeostasis [2], while ghrelin, secreted from gastric mucosa, stimulates hunger in response to fasting [8]. Given that leptin and ghrelin have regulatory roles in maintaining the reproductive capacity and initiating puberty, it is conceivable that they have a dynamic relationship with female sex hormones [2]. Despite the general agreement that leptin is negatively associated with EI and appetite [9] with respect to the MC, studies have reported conflicting results. That is, prior research has reported higher leptin levels coinciding with ovulation [10] and during the luteal phase [11], whereas, other studies have failed to observe a significant effect of the MC on leptin [12–14]. Studies examining the effects of MC on ghrelin have not observed a significant fluctuation [15], although ghrelin concentrations have been negatively associated with daily EI [16]. Finally, a possible factor that may affect EI is food cravings, which can be affected by the MC phase [2].

The concept of energy availability (EA) is defined as dietary EI minus the energy expended due to exercise. As such, EA is the amount of dietary energy remaining after exercise training for all other physiological processes that contribute to maintaining homeostasis in the body [17]. Adequate EA is, of course, required in order to maintain hormonal function, whereas the MC is an indicator of energy balance in women who are not pregnant, nursing or using hormonal contraceptives [18]. Metabolic hormones have been shown to be sensitive to changes in EA in athletes [19], however, the time-course of the changes in EA and metabolic hormones, as well as the possible effect of sex hormones on these associations is still somewhat unclear.

Studies examining EI, EA, EEE, and concentrations of metabolic hormones across the MC or HC are few. As such, the aim of this study was to investigate changes in self-reported EI, EA, macronutrient intake, as well as metabolic hormones, leptin, ghrelin, insulin, and glucose concentrations, across the MC in recreational female athletes using or not using hormonal contraceptives. Additionally, the associations between measured variables were examined.

## 2. Materials and Methods

### 2.1. Participants

Healthy women, age 18–40 years, were recruited by advertisements in social media and the local newspaper. Participants filled in a health questionnaire and Low Energy Availability in Females Questionnaire (LEAF-Q) prior to participation in the study [20]. Inclusion criteria required that participants be recreationally physically active (strength training three times·week^−1^ and endurance training three times·week^−1^) with a BMI of 18–25 kg·m^−2^ and LEAF-Q score < 8. Participants were excluded if they were pregnant or lactating, if they had conditions affecting the ovarian function, amenorrhea, endocrine disorders or chronic diseases or if they were taking medication that may affect exercise responses. All participants reported that they did not smoke, were free from injury, and were not using any medications. Each subject was informed of the potential risks and discomforts associated with the measurements, and all of the subjects gave their written informed consent to participate. The study was conducted according to the Declaration of Helsinki, and the Ethics Committee of the University of Jyväskylä (22 October 2018), approved the study.

A total of 33 women were enrolled in the study. Five participants dropped out prior to the completion of the study due to personal reasons or schedule conflicts. Four more participants were excluded from the analysis due to a lack of information provided in their personal dietary or training logs. Data were ultimately analyzed and are presented for n = 24. Descriptive data (gathered at bleeding (menses/withdrawal bleed)), including participant characteristics are presented in Table 1. Participants were either eumenorrheic and had not used an HC for at least 1 year (NHC = 15) or had used a monophasic contraception with combined synthetic estrogen and progestin HC for at least 1 year (CHC, n = 9). The data presented are part of a larger endogenous and exogenous hormone and performance in women (MEndEx) study.

### 2.2. Study Design

Each participant visited the laboratory four times. In the NHC group, participants visited the laboratory during bleeding (BLE, day 2–4 of the participant’s MC), mid-follicular phase (FP, 7–11 days from the onset of bleeding), ovulation (OVU, determined from the urine test, see below), and mid-luteal phase (LP, 7 days after ovulation). The CHC participants visited the laboratory at the end of inactive phase (non-pill/placebo, bleeding), twice during the active pill phase separated by 7 days, and at the beginning of the inactive (non-pill/placebo) phase. The phase of the MC or HC cycle in which testing commenced was randomized. Data are presented such that the phases of the MC and HC were “matched“ at bleeding. Procedures were performed according to the current recommendations for best practice [21]. Ovulation was identified using a daily urine test completed by the participant at home starting mid-FP to identify the LH surge (Dipro, LH Ovulation Strip, Aidian Oy, Finland). Ovulation was detected in all the NHC participants and MC phases in NHC were retrospectively confirmed by the analysis of serum hormones as described in Section 2.5.

### 2.3. Body Composition

Anthropometric measurements were completed in the morning after 12 h of fasting. The height of the participants was measured with a wall-mounted stadiometer. The body mass and body composition were measured using bioimpedance (Inbody 720, Biospace Co., Seoul, Korea).

### 2.4. Nutrition, Energy Intake, and Energy Availability

Participants were instructed to maintain their typical diet throughout the study and were instructed to continue eating as they normally would, ad libitum. Participants completed 72-h dietary and training logs starting from each laboratory visit. Written and verbal instructions were given to ensure accurate record keeping. The dietary logs were analyzed for energy and macronutrient intake using the software (Fineli, National Institute for Health and Welfare, Helsinki, Finland). Training logs were analyzed for exercise energy expenditure (EEE) using metabolic equivalent of task (MET) values for different activities [22]. EA was estimated as EI minus EEE and expressed in kcal·kg fat-free mass (FFM)^−1^·day (d)^−1^ [17]. Participants reported food cravings, assessed dichotomously as “yes“ or “no“, as part of the dietary log. If the participants answered “yes“, they were asked to record specific food cravings and the actual food item(s) craved. The number of “yes” answers was calculated. “Yes” included sweet, salty, soda drinks, and experiencing more hunger than usual. “No” included mentions of absence of cravings or the absence of notes on cravings.

### 2.5. Venous Blood Samples

Blood samples were collected in the morning (7:00–9:00 a.m.) after a 12 h overnight fast. Participants were instructed to abstain from strenuous physical activity for 24 h before the blood samples were taken. Venous blood samples were drawn from an antecubital vein using standard procedures and the blood was transferred into serum and EDTA tubes (Venosafe, Terumo, Belgium). The serum samples were held for 15 min at room temperature before being centrifuged for 10 min at 2000× *g* (Megafuge 1.0 R, Heraeus, Germany). The serum was separated and immediately frozen at −80 °C for later analysis. Leptin was assessed with the Biovendor Human Leptin ELISA. Total ghrelin was assessed with the Biovendor Human Ghrelin Easy Sampling ELISA from plasma after incubation at room temperature for 2 h. The assay sensitivity for ghrelin was 10 mg·L^−1^. Other hormonal analyses were performed using chemical luminescence techniques (Immulite 2000, Siemens Healthcare Diagnostics, Camberley, UK) with an assay sensitivity of 55.0 pmol·L^−1^ for E2, 0.3 ng·mL^−1^ for P4, 0.2 ng·mL^−1^ for leptin, 1.5 mmol·L^−1^ for T3, 10 ng·L^−1^ for ghrelin, 2 mIU·l^−1^ for insulin, and 0.1 nmol·L^−1^ for glucose. Inter-assay coefficients of variation (CV) were 6.7% for E2, 9.7% for P4, 4.2% for leptin, 8.1% for T3, 6.8% for ghrelin, 5.1% for insulin, and 1.4% for glucose.

### 2.6. Statistical Analyses

Statistical analyses were conducted using SPSS Statistics 24 (IBM, Armonk, NY, USA). Results are reported as mean ± SD. Due to the small sample size, nonparametric tests were used. A Mann-Whitney-U test was used to examine baseline differences between groups, while Friedman’s ANOVA was used to analyze a main effect for time. A Wilcoxon signed-rank test was used to complete pair-wise comparisons between time points. Between group differences in food cravings were examined using the Chi Square test. The related-samples Cochrans Q test assessed the effect of MC and HC phase on cravings. Associations between hormones and dietary intake were examined with Spearman’s correlation. Statistical significance was defined as *p* < 0.05.

## 3. Results

### 3.1. Hormonal Fluctuations

Concentrations of analyzed hormones for each phase are presented in Table 2. As expected, E2 and P4 fluctuated significantly in NHC and remained stable in CHC. In NHC, E2 was significantly higher at FP, OVU, and LP than at BLE (*p* = 0.006, *p* = 0.005, *p* = 0.001, respectively). Concentrations of E2 were higher in NHC than in CHC at FP/active1 (*p* = 0.002), OVU/active2 (*p* = 0.004), and at LP/inactive (*p* < 0.001). In NHC, P4 was higher at OVU and FP than BLE (*p* = 0.030 and *p* = 0.001), as well as being higher at LP and FP than BLE *(p* = 0.001 and *p* = 0.006). Concentrations of P4 were higher in NHC than in CHC at OVU/active2 (*p* = 0.017), and at LP/inactive (*p* = 0.003).

In NHC leptin increased significantly between BLE and OVU (*p* = 0.030) as well as between FP and OVU (*p* = 0.022), however, no group differences were observed across phases between NHC and CHC.

**Figure 1.**
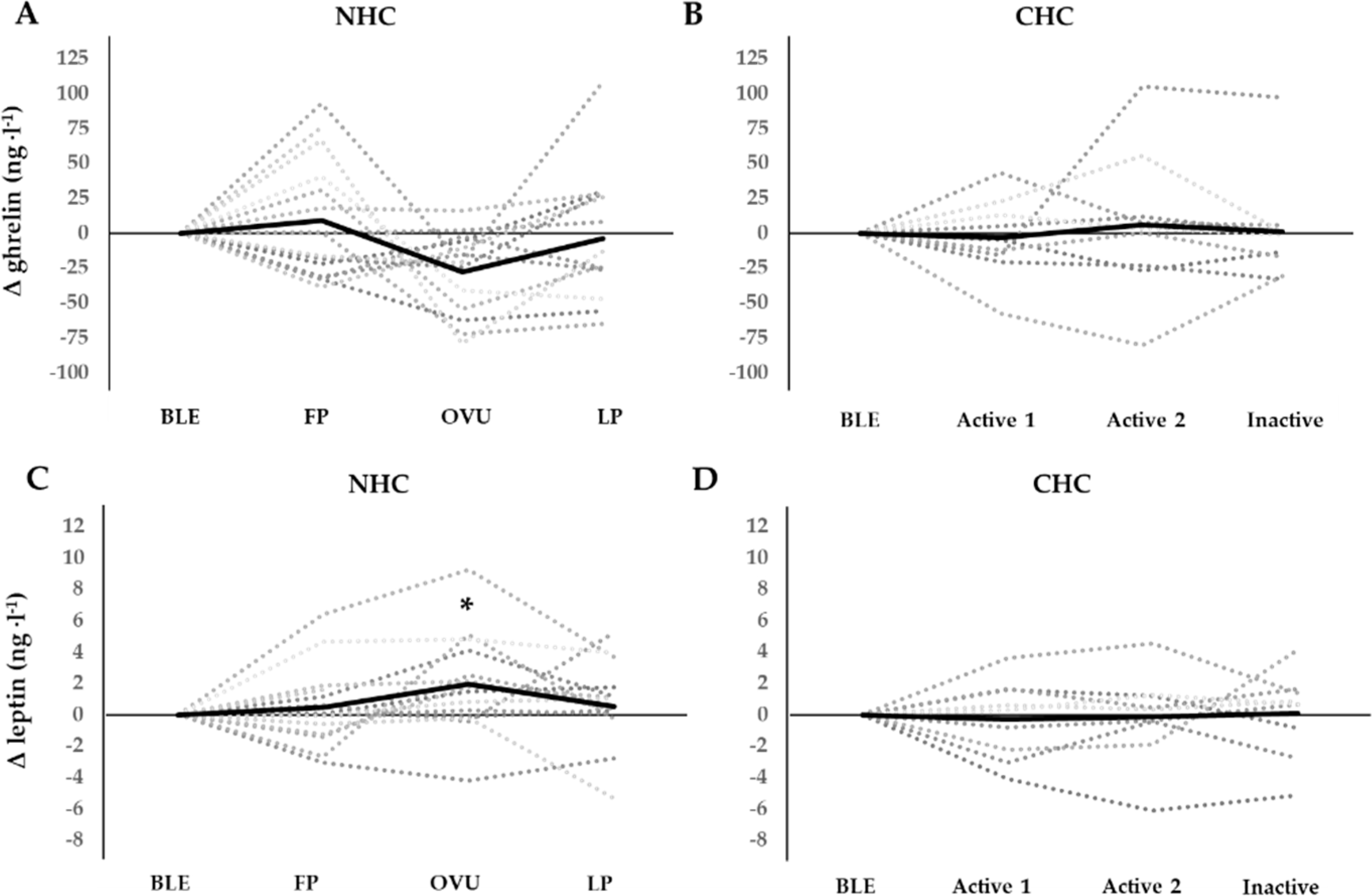
Individual profiles for changes in ghrelin and leptin concentrations across MC phases in eumenorrheic women not using hormonal contraception (NHC, panels (**A**,**C**)) and across HC phases in women using hormonal contraception (CHC, panels (**B**,**D**)). Leptin was significantly elevated from bleeding to ovulation and follicular phase to ovulation in NHC. * = *p* < 0.05. BLE : bleeding; FP: mid follicular phase; OVU: ovulation; LP: mid luteal phase.

Ghrelin, insulin, T3, and glucose remained stable over phases in both NHC and CHC. No group differences were observed between NHC and CHC for ghrelin, insulin, and glucose, while T3 was higher in CHC at BLE, OVU/active2, and LP/inactive. Individual profiles of leptin and ghrelin concentrations across MC and HC phases are presented in Figure 1.

### 3.2. Nutritional Intake and Energy Avalability

Table 3 summarizes the energy and macronutrient intake analyzed from the dietary logs as well as the energy expenditure analyzed from training logs. The mean EI, EEE, and EA were similar between phases and there were no significant differences observed in EI or macronutrient intake (CHO, PROT, and FAT) over MC or HC. At BLE and LP/inactive, however, statistical trends for higher CHO in CHC in comparison to NHC were observed (*p* = 0.068 and *p* = 0.069, respectively). EA was significantly higher in CHC than NHC at LP/inactive (*p* = 0.017). Furthermore, there was a trend for higher EA at FP/active 1 (*p* = 0.052), and OVU/active2 (*p* = 0.063).

### 3.3. Body Mass and Cravings

**Figure 2.**
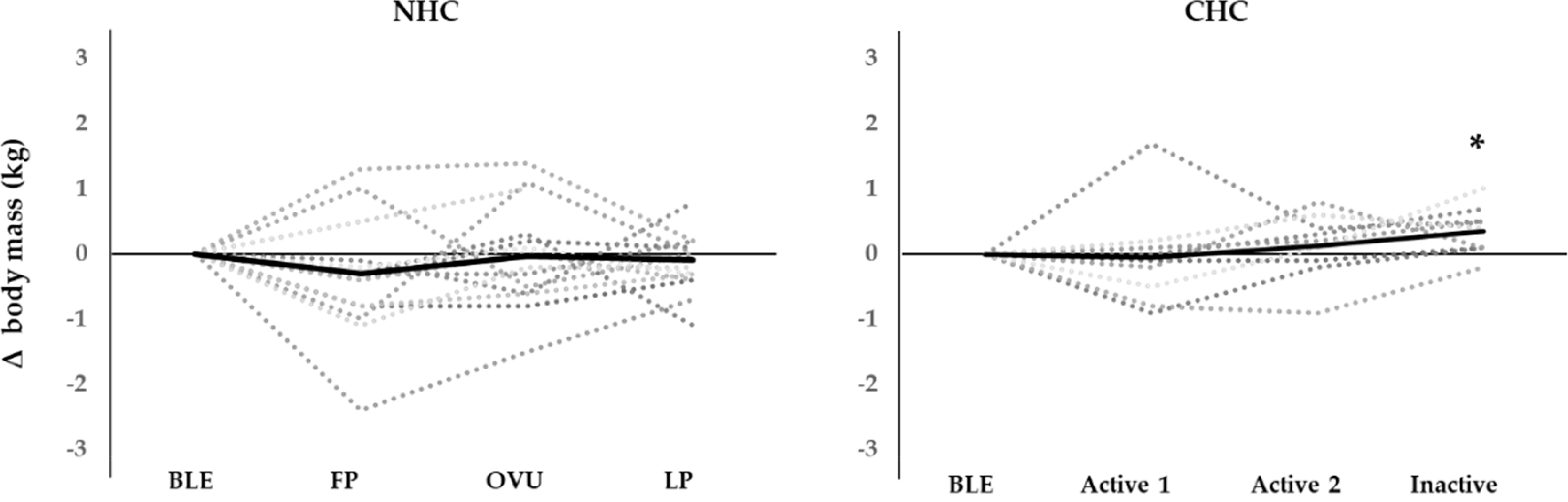
Individual profiles for changes in body mass across MC phases in eumenorrheic women not using hormonal contraception (NHC) and across HC phases in women using hormonal contraception (CHC). BLE: bleeding; FP: mid follicular phase; OVU: ovulation; LP: mid luteal phase. Body mass was significantly elevated from bleeding to inactive in CHC. * = *p* < 0.05.

In NHC, a trend for body mass fluctuation was observed (*p* = 0.055), however, post-hoc tests did not reveal any significant differences between MC phases. In CHC, the body mass fluctuated significantly (*p* = 0.017) with post hoc testes revealing a small, but statisticanlly significant increase (0.4 ± 0.1 kg, *p* = 0.028) from BLE to the inactive phase. Individual profiles of body mass across MC and HC phases are presented in Figure 2.

In NHC, 19%, 25%, 25%, and 50% of the participants reported food cravings at BLE, FP, OVU, and LP, respectively. Whereas, in CHC, 67%, 78%, 56%, and 44% of the participants reported food cravings at BLE, active1, active2, and inactive, respectively. Interestingly, NHC had significantly fewer cravings than CHC at BLE (*p* = 0.022), and at FP/active1 (*p* = 0.015). No significant with-in group fluctuations in cravings across the MC or HC were observed.

### 3.4. Associations

EI and EA were not associated with metabòlic hormones or sex hormones. When NHC and CHC were pooled, significant negative associations were observed between ghrelin and leptin at BLE (*ϱ*= −0.465 *p* = 0.022), at FP/active1 *(**ϱ* = 0.507, *p* = 0.011), at OVU/active2 *(**ϱ* = −0.631, *p* < 0.00), and at LP/inactive (*ϱ* = −0.428, *p* = 0.042). As expected, body fat % correlated with average leptin (*ϱ* =0.531, *p* = 0.011).

## 4. Discussion

The purpose of this investigation was to examine the effects of MC and HC phase (endogenous and exogenous hormones) on EI and metabolic hormones in recreational athletes. Of the measured hormones associated with metabolism, only leptin fluctuated significantly during the MC in eumenorrheic women. These alterations in leptin concentrations, however, did not correlate with changes in E2 or P4. In women using combined HC, the HC phase did not alter metabolic hormones, while sex hormone concentrations also remained stable. No significant alterations in EI or EA were observed in either group with respect to the MC or HC phase. These findings suggest that neither MC nor HC phase, on average, alters ad libitum EI, however, it should be emphasized that large inter-individual differences were observed within our data.

We demonstrated a small but significant elevation in leptin during OVU compared to BLE and FP, a finding that is in line with previous research [10,23–25] although it is important to note that this finding is not consistent [26–28]. Ajala et al. (2013) suggested that several factors determining leptin expression may account for varying concentrations across the MC phases, including the regulatory properties of ovarian steroid hormones [24]. Lin et al. (1999), on the other hand, reported no significant associations between E2, P4, and leptin in any MC phase [27]. Although some studies have suggested that leptin parallels concentrations of progesterone [10,23], we did not observe this phenomena. Higher concentrations of leptin around OVU and during the LP, however, may be supported by the documented existence of leptin receptors in ovaries, follicles, and the corpus luteum [29]. The ability to compare data between studies is limited by methodological variances such as differences in blood assays and procedures used for the verification of MC phases. In the present study, procedures for MC phase identification were performed according to current recommendations for best practice [21]. Meanwhile, it is notable that relatively large inter-individual variation in hormonal concentrations, as reported in previous studies, was also present in our study. As expected, we found that leptin was associated with the fat % of the participants. Nevertheless, regardless of changes in body mass across the MC, the fat mass and EI of subjects remained statistically unaltered across the MC. It can be assumed that the significant change in leptin in NHC were not due to dramatic energy imbalances. Hence, changes in leptin might be explained by other mechanisms, such as the above-mentioned post-ovulatory changes. A variation in leptin was not observed in CHC.

There was a statistical trend for phase in ghrelin in NHC, where the lowest concentrations were observed at OVU. A similar non-significant decrease at mid-cycle was observed by Šramkóvá et al. (2015) [30]. As ghrelin and leptin have opposite roles in the control of satiety, this trend warrants more research. Although no studies, to date, have demonstrated a relationship between ghrelin and the MC in healthy women, an interplay between ghrelin and sex hormones cannot be completely ruled out. Exogenous E2 and P4, in the form of HC, increased ghrelin in women suffering from polycystic ovarian syndrome [31], while exogenous E2 has been shown to increase ghrelin in postmenopausal women [32]. Interestingly, HC use has not been demonstrated to influence ghrelin in healthy young women [33], a finding in agreement with the results of this study. Again, it is essential to consider the methodological discrepancies between studies. In line with our methods, some researchers have assessed total ghrelin [30,31], while others have assessed acylated ghrelin (AG) and unacylated ghrelin (UnAG) separately [15]. AG seems to have greater significance with regards to appetite stimulation, while total ghrelin reflects mainly UnAG, representing as much as 90 % of total plasma concentration [34]. Due to the sample collection in this study, total ghrelin was assessed, thus our results cannot be directly compared to all previous findings.

A phase effect of the MC or HC on fasting insulin, glucose or T3 was not observed, which is mostly in agreement with the existing literature. Only insulin has been shown to vary across the MC [35,36]. It is noteworthy to consider that the majority of the studies investigating the relationship between glucose metabolism and the MC have tested insulin sensitivity and glucose tolerance, with very few assessing fasting concentrations, as in the present study. Interestingly, T3 was significantly higher in CHC compared to NHC at all measurement points except at FP/active1. Although the difference between EA was statistically significant only at LP/inactive, EA was slightly higher in CHC than NHC throughout the investigation, which might explain the differences between groups in T3 concentrations in [37]. Indeed, HC use appears to increase T3 concentrations [38].

On average, EI, EEE, and EA remained relatively stable over the MC and HC. Furthermore, no changes in macronutrient preference was detected between phases or groups. As such, our results do not offer compelling evidence to indicate that dietary intake changes markedly over the course of a single MC or HC. Therefore, this study suggests that eumenorrheic recreationally active women are not more vulnerable to MC phase based perturbations in their habitual eating behavior compared to their counterparts that have a more stable hormonal milieu due to HC use. Previous laboratory-based interventions and cross-sectional observations of low EA have reported effects on several metabolic hormones including decreased triiodothyronine (T3), leptin, and insulin [39,40]. It is important to acknowledge that energy requirements are not only affected by resting metabolic rate and dietary intake, but also by EEE, and thus EA may better describe the nutritional status of highly active participants than EI alone. Considering the present results, researchers should not worry about the inclusion of women in research due to potential changes in EI, EEE or EA over the MC or HC, although it is worth remembering that a significant fluctuation may occur on an individual level. Likewise, it may be important to acknowledge that in a physically active population, such as the one investigated in this study, the use of HC does not appear to be a significant factor modulating leptin and ghrelin concentrations, although T3 concentrations, on average, were higher in women using HC.

We observed an increase in body mass from BLE to the inactive phase in CHC. Meanwhile, no significant fluctuations were detected in NHC. Again, it is notable that relatively large inter-individual variations were observed in both groups. Previous studies examining changes in body mass across the HC phase are sparse, and to our knowledge there are not any studies that have reported a similar increase in body mass during the HC cycle [41,42]. Meanwhile, most previous studies investigating body mass changes across the MC are in line with our study and indicate a lack of evidence to support a significant fluctuation of body mass across the MC in athletic women [41,43,44]. Nevertheless, it has been suggested that body mass increases from the FP to the LP in athletic women, which has been explained by fluid retention caused by higher aldosterone concentrations [45] or increased food consumption in the LP [46]. Nevertheless, the effect of the MC and HC on body mass has not been fully elucidated [47].

Our study demonstrated that women in CHC experience more cravings compared to NHC during the first half of the cycle (BLE; active1/FP), as well as a tendency for women in NHC to report more cravings at LP compared to other phases. These findings must be interpreted with caution, given the inconsistent pattern they exhibit. There is some evidence suggesting that MC-related cravings are often experienced in the LP [48] reflecting the orexigenic effects of progesterone [2]. However, previous studies have not observed any differences in cravings between HC users and non-users [49]. It is crucial to note that the concept of cravings comprises a sum of complex factors including social and psychological dimensions along with hormones, thus these findings do not allow for strong conclusions. Taken together, the present study demonstrated between-group differences in food cravings and T3 between MC and CHC. These results may suggest a minor effect of HC use on cravings and EA, however further exploration with a larger study population is needed.

The current study had several limitations, including self-reporting of dietary and training information, as well as the limited number of participants. However, nearly all studies using questionnaires suffer from such constraints. It is well known, that investigation of EI is sensitive to numerous confounding factors, such as personality traits [50] and eating behaviors [51]. Participants’ conventional eating patterns and attitudes towards nutrition may have obscured associations between the MC or HC cycles and dietary intake. Discussion regarding the influence of EI, macronutrient intake, and EA on training responses and/or performance, where the possible influence of MC and HC phase are taken into consideration may be warranted, however, this goes beyond the scope of the present article. We acknowledge these shortcomings, but also emphasize the strengths of the present study. To our knowledge, there are no studies examining the association between EA and metabolic hormones with respect to the MC and HC phase, as such, this pilot study appears to be novel in this area of research. All of the participants were highly motivated to provide researchers with accurate information and the research team took great care to interact with the participants throughout the study by encouraging their full and complete compliance with the protocols. Finally, our study included four time-points rather than the usual two used in many studies. We also incorporated a prospective determination of MC phases, as well as retrospective confirmation of both MC and HC phases according to the current recommendations for best practice [21].

## 5. Conclusions

The MC phase can have a small but significant effect on leptin concentrations although neither MC nor HC phase appeared to affect other metabolic hormones measured in the present study. Furthermore, EI, EEE, and EA did not change over the MC or HC phase suggesting that MC or HC phase does not alter ad libitum EI or EA in recreational athletes. This finding also indicates that monitoring of EI, EEE, and EA in each phase of the MC or HC may not be necessary in e.g., longitudinal training studies. It should be acknowledged that a large inter-individual variation within our data may limit the interpretation of our results, although this variation also underscores the importance of considering the individual rather than group means in practice.

## Figures and Tables

**Table 1.** Participant information. NHC: Women not using hormonal contraception; CHC: Women using hormonal contraception; LEAF-Q: Low Energy Availability in Females Questionnaire. Results are presented as mean ± SD.

	NHC (n = 15)	CHC (n = 9)

Age (years)	26 ± 4	23 ± 2
Body mass (kg)	67.6 ± 6.5	61.0 ± 4.3
Height (m)	1.67 ± 0.06	1.70 ± 0.06
Body fat (%)	22.1 ± 6.7	19.5 ± 2.8
LEAF-Q (score)	4.5 ± 2.1	5.7 ± 1.8

**Table 2.** Serum hormone and glucose concentrations across the measurement points BLE: bleeding; FP: mid follicular phase; OVU: ovulation; LP: mid luteal phase; E2: Estradiol; P4: Progesterone; T3: Tri-iodothyronine. Values are presented as mean ± SD.

	Group	BLE	FP/Active1	OVU/Active2	LP/Inactive	Phase

E2 (pmol·L^−1^)	NHC	290 ± 140	560 ± 390 **	690 ± 500 **	650 ± 240 **	*p* < 0.001
	CHC	300 ± 270	190 ± 140 ^aa^	220 ± 230 ^aa^	190 ± 110 ^aaa^	*p* = 0.435

P4 (nmol·L^−1^)	NHC	2.0 ± 1.7	1.0 ± 0.5	4.1 ± 2.7 *^,++^	15.0 ± 8.9 **^,++,##^	*p* = 0.001
	CHC	1.1 ± 0.5	1.0 ± 0.5	1.1 ± 1.0 ^a^	1.2 ± 1.0 ^aa^	*p* = 0.239

Leptin (ng·L^−1^)	NHC	6.8 ± 4.0	7.2 ± 5.4	8.5 ± 6.2 *^,+^	7.8 ± 5.2	*p* = 0.014
	CHC	8.3 ± 7.4	8.0 ± 7.8	8.2 ± 7.6	8.4 ± 6.5	*p* = 0.706

Ghrelin (ng·L^−1^)	NHC	238 ± 72	247 ± 68	210 ± 75	228 ± 74	*p* = 0.089
	CHC	211 ± 106	208 ± 101	217 ± 138	212 ± 136	*p* = 0.352

Insulin (mIU·L^−1^)	NHC	2.8 ± 1.7	2.5 ± 1.7	3.8 ± 3.6	3.0 ± 2.6	*p* = 0.183
	CHC	2.9 ± 2.9	3.4 ± 2.8	4.2 ± 3.0	2.9 ± 2.4	*p* = 0.376

T3 (pmol·L^−1^)	NHC	4.9 ± 0.4	4.6 ± 0.7	4.9 ± 0.6	5.0 ± 0.7	*p* = 0.119
	CHC	5.6 ± 0.7 ^aa^	5.4 ± 0.9	5.6 ± 0.9 ^a^	6.2 ± 1.0 ^aa^	*p* = 0.062

Glucose (nmol·L^−1^)	NHC	5.0 ± 0.3	5.1 ± 0.4	4.9 ± 0.5	4.9 ± 0.4	*p* = 0.384
	CHC	4.8 ± 0.4	4.8 ± 0.5	4.9 ± 0.5	4.9 ± 0.3	*p* = 0.519

Significant difference from BLE

* = *p* < 0.05 and

** = *p* <0.01.

Significant difference from FP

+ = *p* < 0.05 and

++ = *p* < 0.01.

Significant difference from OVU

## = *p* < 0.01.

Significant difference from NHC

a = *p* < 0.05

aa = *p* < 0.01, and

aaa = *p* < 0.001.

**Table 3.** Nutritional intake and energy availability. BLE: bleeding; FP: mid follicular phase; OVU: ovulation; LP: mid luteal phase; EI: Energy intake; EEE: Exercise energy expenditure; EA: Energy availability; CHO: Carbohydrate intake; PROT: Protein intake; FAT: Fat intake across MC and HC phases. Values are presented as mean ± SD.

	Group	BLE	FP/Active1	OVU/Active2	LP/Inactive	Phase

EI (kcal·day^−1^)	NHC	2340 ± 660	2340 ± 540	2280 ± 510	2270 ± 370	*p* = 0.825
	CHC	2770 ± 500	2470 ± 510	2660 ± 710	2510 ± 380	*p* = 0.081

EEE (kcal·day^−1^)	NHC	325 ± 157	342 ± 109	372 ± 170	361 ± 199	*p* = 0.099
	CHC	248 ± 117	326 ± 94.8	251 ± 90	251 ± 90	*p* = 0.591

EA (kcal·kgFFM^−1^·day^−1^)	NHC	40.0 ± 11.1	39.9 ± 11.1	35.9 ± 9.0	37.6 ± 7.2	*p* = 0.465
	CHC	42.9 ± 9.6	51.7 ± 11.4	49.4 ± 17.4	45.5 ± 5.4 ^a^	*p* = 0.054

CHO (g·day^−1^)	NHC	255 ± 80	260 ± 77	247 ± 67	250 ± 55	*p* = 0.897
	CHC	310 ± 60	273 ± 61	300 ± 91	293 ± 44	*p* = 0.506

PROT (g·day^−1^)	NHC	112 ± 40	107 ± 30	110 ± 27	105 ± 31	*p* = 0.873
	CHC	118 ± 39	109 ± 27	109 ± 30	110 ± 36	*p* = 0.072

FAT (g·day^−1^)	NHC	86 ± 27	86 ± 33	85 ± 21	83 ± 19	*p* = 0.992
	CHC	105 ± 30	91 ± 29	100 ± 34	84 ± 13	*p* = 0.102

Significant difference from CHC

a = *p* < 0.05.
